# AI-enhanced soil classification with incomplete CPT data for offshore wind farm

**DOI:** 10.1038/s41598-026-46356-6

**Published:** 2026-03-30

**Authors:** Cheng-Yu Ku, Ting-Yuan Wu, Chih-Yu Liu, Wen-Yang Hsu

**Affiliations:** 1 Department of Harbor and River Engineering, National Taiwan Ocean University, Keelung, 202301 Taiwan; 2 Center of Excellence for Ocean Engineering, National Taiwan Ocean University, Keelung, 202301 Taiwan

**Keywords:** Offshore wind farm, Soil classification, Cone penetration test, Artificial intelligence, Machine learning, Engineering, Environmental sciences, Mathematics and computing

## Abstract

Accurate soil classification is fundamental to offshore wind farm foundation design, yet conventional cone penetration test (CPT) based methods often require complete datasets that are costly and challenging to obtain in offshore environments. This study presents an artificial intelligence (AI) enhanced framework for soil classification based on the Robertson Classification, with a particular emphasis on robustness under incomplete CPT data. A comprehensive synthetic CPT database comprising 229,808 samples was generated using both uniform and statistically distributed sampling strategies to represent a wide range of realistic soil conditions. Among the four evaluated machine learning models, the random forest model achieved the best performance, with an R² of 0.99 and a classification accuracy of 92.53%. Simulations of missing CPT input parameters reveal that reliable predictions can be maintained even under incomplete data scenarios. Feature importance indicates that cone tip resistance (*q*_*c*_), sleeve friction (*f*_*s*_) and effective stress (*σ’*_*v*_), are the dominant factors governing soil classification. Prediction uncertainty using Monte Carlo simulations shows model performance within a 95% confidence interval. Overall, the proposed AI-enhanced framework provides a robust and practical solution for CPT-based soil classification using incomplete datasets for offshore wind farm geotechnical design.

## Introduction

Soil classification constitutes a fundamental component of geotechnical engineering, as it provides a systematic framework for interpreting subsurface conditions and predicting soil behavior relevant to the design of foundations, offshore structures, embankments, and earth-retaining systems (Terzaghi, 1996;^1^. In offshore wind farm developments, accurate characterization of seabed soils is particularly critical because foundation performance, installation feasibility, and long-term structural stability are strongly governed by soil stratigraphy and mechanical properties (Wang et al., 2022;^2,3^. Among traditional classification frameworks, the unified soil classification system (USCS) remains one of the most widely adopted standards for engineering communication and design^4–6^. The USCS classifies soils based on grain-size distribution and plasticity characteristics derived from laboratory tests, such as sieve analysis and Atterberg limits^7^; Ching & Farahbakhsh, 2025). Despite its widespread acceptance, USCS classification relies heavily on high-quality sampling and laboratory testing, which can be challenging, time-consuming, and costly in offshore environments, particularly at large water depths^8,9^.

To overcome these limitations, in situ testing methods, most notably the cone penetration test (CPT), have become central to offshore soil investigation. CPT offers continuous, repeatable profiling of subsurface conditions and is especially well suited for marine applications. CPT-based soil classification systems translate measured parameters, such as pore pressure, sleeve friction, and cone tip resistance, into soil behavior type indices that approximate traditional classifications, including those of the USCS^10,11^; Ching, & Farahbakhsh, 2025). Numerous empirical and semi-empirical CPT charts have been proposed to bridge CPT measurements with USCS soil groups, enabling rapid interpretation without extensive laboratory testing^12,13^. These CPT-based approaches are now widely used in offshore oil, gas, and wind energy projects for preliminary site characterization and foundation design^14,15^. However, conventional CPT-based classification methods generally assume the availability of complete, high-quality datasets and rely on deterministic or semi-empirical boundaries, which may limit their applicability under imperfect field conditions^16–18^. In practice, offshore site investigations are often constrained by water depth, weather windows, equipment limitations, and project budgets^12^. As a result, CPT datasets may contain missing, noisy, or partially unreliable measurements, reducing the effectiveness of traditional classification approaches and increasing uncertainty in engineering interpretation^19^; Yetginer-Tjelta et al., 2022). These challenges highlight the need for advanced data-driven techniques capable of extracting meaningful patterns from incomplete datasets while preserving consistency with established geotechnical knowledge^5,12,16^. Recent advances in artificial intelligence (AI) and machine learning (ML) provide a powerful complement to traditional soil classification methods^5^. Previous studies have demonstrated that ML models can enhance prediction accuracy, reduce interpretation subjectivity, and provide rapid decision support in site characterization and foundation design^20^. Integrating ML with established CPT-based soil classification frameworks therefore offers a promising pathway to improve robustness and reliability, especially in offshore environments where data completeness cannot always be guaranteed^21^.

Recent studies have increasingly applied probabilistic and machine-learning approaches to geotechnical interpretation and liquefaction assessment using CPT and related in-situ data. Bayesian and data-driven frameworks have been developed to incorporate soil classification, parameter uncertainty, and model uncertainty into engineering decision-making. These studies demonstrate the growing integration of statistical learning and geotechnical mechanics, which motivates the development of robust data-driven soil behavior classification methods^22,23^.

Relevant literature on probabilistic CPT-based soil profiling and uncertainty-aware soil behavior classification has been reviewed and incorporated into the Introduction. These studies consider uncertainties associated with soil behavior type (SBT) chart boundaries and inherent variability in CPT measurements. Previous research has addressed uncertainty in CPT interpretation using probabilistic and statistical frameworks^24,25^. The novelty of the present study lies in the fact that, while existing probabilistic approaches improve reliability when complete CPT data are available, the proposed framework focuses on soil behavior classification under incomplete CPT measurements. Therefore, this work provides a complementary contribution by addressing information deficiency rather than solely measurement uncertainty. This study develops and evaluates an AI-enhanced soil classification framework tailored for offshore wind farm applications, with particular emphasis on performance under incomplete CPT data conditions. The objective is to demonstrate that reliable soil classification can be achieved even when certain CPT parameters are missing, thereby reducing reliance on labor-intensive offshore investigations while maintaining classification accuracy^26^.

### Robertson classification

The Robertson Classification is an empirical soil behavior classification framework widely applied in geotechnical engineering for interpreting subsurface conditions based on CPT data. Originally proposed by Robertson and Campanella and subsequently refined by Robertson^11^, this method characterizes soil behavior through CPT-derived measurements, including pore water pressure response, sleeve friction, and cone tip resistance^10,27^. The Robertson Classification provides a behavior-based interpretation of soils that integrates both mechanical and hydraulic responses, making it particularly suitable for site characterization in heterogeneous deposits. The method has been extensively adopted in offshore, onshore, and seismic geotechnical investigations due to its efficiency and repeatability.

**Fig. 1:**
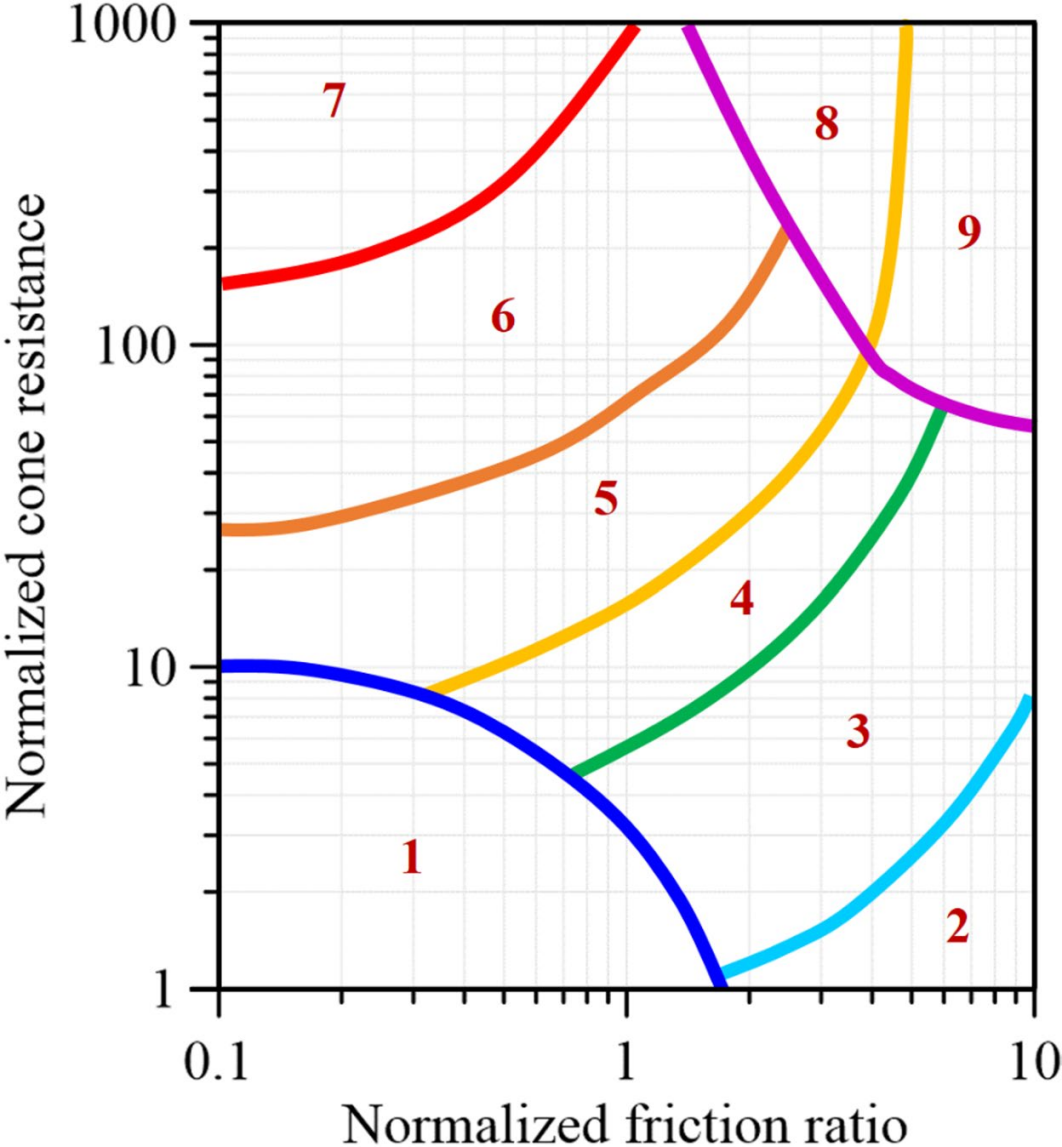
Contours of the soil behavior type index^11^.

The Robertson Classification evaluates soil behavior primarily through CPT-derived input parameters, which are commonly expressed in normalized form to reduce the influence of overburden stress. These parameters collectively describe soil strength, stiffness, and drainage characteristics, forming the basis for soil behavior classification within the Robertson framework. Figure 1 illustrates the contours of the soil behavior type index as proposed by Robertson^11^. This contour-based representation allows for a more refined interpretation compared to discrete soil classification zones and provides a quantitative basis for subsequent analysis. The Robertson Classification establishes a direct link between CPT measurements and soil behavior interpretation, enabling consistent soil classification during site investigation and supporting geotechnical design and analysis^28^.

Robertson^11^, SBT chart uses normalized cone resistance (*Q*_*tn*_) on the vertical axis. In this study, the full stress normalization procedure was adopted. The stress exponent *n* was not assumed as a constant but was calculated following Robertson^11^ as a function of the soil behavior index and effective vertical stress^29^:

1 $$n=0.381{I_c}+0.05\frac{{{{\sigma ^{\prime}}_v}}}{{100}} - 0.15$$

where *I*_*c*_ is the soil behavior type index, ${\sigma ^{\prime}_v}$ is the effective vertical stress,. The normalized cone resistance *Q*_*tn*_ was then computed using this stress exponent. Therefore, the SBT chart in this study is consistent with the Robertson^11^ formulation rather than a simplified *Q*_*t*1_ normalization^30^.

CPT interpretation relies on a set of interrelated measurements that together describe soil behavior and form the basis of the Robertson soil classification framework. The primary measurement is tip resistance (*q*_*c*_), which represents the resistance of the soil to penetration by the cone tip and reflects the combined influence of soil strength, stiffness, and in-situ stress conditions^31,32^. High *q*_*c*_ values are typically associated with dense sands, gravels, or overconsolidated soils, while low values indicate soft clays or loose deposits. Because tip resistance is strongly stress-dependent, it is commonly normalized to allow meaningful comparison across depths and geological settings, making it a cornerstone parameter in defining soil behavior types. Complementing tip resistance is sleeve friction (*f*_*s*_), which measures the frictional resistance acting along the cylindrical sleeve behind the cone tip. Sleeve friction is particularly sensitive to soil plasticity, mineralogy, and adhesion, with cohesive soils generally exhibiting higher friction relative to qc than granular soils. The friction ratio, defined as the ratio of fs to *q*_*c*_, is therefore a key indicator used to distinguish clay-dominated from sand-dominated behavior and to refine soil behavior interpretation^33,34^.

Pore water pressure, most commonly measured at the cone shoulder as *u*_*2*_, provides insight into the soil’s drainage response during penetration. Low-permeability soils such as clays tend to generate significant excess pore water pressures, whereas permeable sands typically show minimal response. The pore pressure signal conveys valuable information about compressibility, consolidation state, and stress history, and its inclusion in normalized CPT parameters greatly improves soil behavior classification, particularly in fine-grained and transitional soils^35^; Golestani & Ahmadi, 2021).

Interpretation of these CPT measurements requires consideration of in-situ stress conditions, beginning with the total vertical stress (*σ*_*v*_), which represents the stress imposed by the overlying soil mass and is calculated from unit weight and depth. Total stress establishes the baseline stress environment influencing cone resistance and is essential for stress normalization, especially in deep deposits (Soleimani & Goudarzy, 2021; Fu et al., 2021). More fundamentally, effective vertical stress (*σ’*_*v*_), defined as the difference between total stress and pore water pressure, governs soil strength and deformation behavior. Since CPT responses are closely linked to effective stress, normalization procedures explicitly incorporate *σ’*_*v*_ to reduce stress-level dependency and to enable consistent classification across normally consolidated and overconsolidated soils^36,37^.

Accurate estimation of static pore water pressure (*u*_0_), representing equilibrium pore pressure prior to testing, is also critical, as it is required to compute excess pore pressure and effective stress. Errors in *u*_0_ estimation can propagate through normalization procedures and compromise soil behavior interpretation, particularly in saturated fine-grained soils^38,39^.

The integration of normalized tip resistance, friction ratio, and pore pressure response ultimately leads to the assignment of a soil behavior type zone. These zones represent the final output of the Robertson Classification and categorize soils according to dominant mechanical and drainage behavior rather than strict lithological boundaries^40,41^.

By synthesizing multiple CPT parameters within a stress-consistent framework, the soil behavior type zones provide a practical and robust means of subsurface characterization that is widely applied in geotechnical site investigation, foundation design, and offshore engineering^42^^[,43^^[,44^.

### Synthetic dataset development

A synthetic dataset was developed to capture the full variability of the key CPT-derived parameters used in the Robertson Classification framework, including cone tip resistance, sleeve friction, pore water pressure response, and stress-related variables, across a wide range of soil conditions. Because comprehensive field datasets that consistently include high-quality CPT measurements are difficult to obtain, particularly for offshore wind farm projects where site investigations are costly, logistically constrained, and often limited in spatial coverage, synthetic data provide an effective and practical approach for systematically exploring parameter interactions and evaluating model performance under controlled conditions. To ensure that the selected parameter ranges are both theoretically sound and representative of realistic geotechnical practice, a systematic review of published CPT-based studies, offshore geotechnical investigations, and relevant technical guidelines was conducted. The resulting parameter bounds and sampling intervals adopted in this study are summarized in Table 1.

Random sampling, together with appropriate statistical distribution assumptions, was applied to generate a large synthetic dataset representing the full range of plausible soil conditions within the Robertson Classification framework. In this study, the synthetic CPT dataset was constructed using two complementary strategies: uniform interval-based sampling of CPT-derived parameters and statistically distributed sampling based on predefined probability distributions. Both sampling approaches were implemented using the input variables summarized in Table 1, incorporating the specified parameter ranges and sampling increments for each CPT-related variable.

**Table 1:** Summary of the input in this study.

Factor	Parameter	Unit	Range	Source
1	Tip resistance (*q*_*c*_)	kPa	0–50,000	Yang^45^,
2	Sleeve friction (*f*_*s*_)	kPa	0–1,000	
3	Pore water pressure (*u*_2_)	kPa	0–500	Snijders and Drijver^46^, Zhang et al.^47^,
4	Total stresses (*σ*_*v*_)	kPa	0–1,000	
5	Effective stresses (*σ’*_*v*)_	kPa	0–500	
6	Static pore water pressure (*u*_0_)	kPa	0–500	
7	Zone	NA	1–9	Robertson^11^,

### Uniform interval–based parameter sampling

For the uniformly spaced sampling scheme, a synthetic CPT-based database was constructed by uniformly distributing each input parameter within its theoretically and empirically reasonable range, as reported in previous CPT-based soil classification studies. The parameter space was discretized using equal intervals, and six CPT-derived variables *q*_*c*_, *f*_*s*_ *u*_*2*_, *σ*_*v*_, *σ’*_*v*_, and *u*_0_, were systematically sampled within the ranges summarized in Table 1, following established CPT practice and published guidelines^45–47^. Specifically, *q*_*c*_ was varied from 0 to 50,000 kPa to represent soil conditions ranging from very soft clays to dense sands and gravels, while *f*_*s*_ ranged from 0 to 1,000 kPa to capture variations in soil plasticity and frictional response. The *u*_*2*_ was assigned values between 0 and 500 kPa, reflecting differences in drainage behavior and soil compressibility. The *σ*_*v*_ and *σ’*_*v*_ were varied within 0–1,000 kPa and 0–500 kPa, respectively, to account for stress-dependent CPT behavior across different depths and geological settings. The 0 was sampled from 0 to 500 kPa to represent a wide range of groundwater conditions. Based on the interaction of these CPT-derived parameters, each synthetic data entry was subsequently mapped to a soil behavior classification zone according to the Robertson Classification framework^11^, with output zones ranging from 1 to 9. Through this uniform combinatorial expansion, a total of 117,649 synthetic CPT data records were generated. The resulting dataset spans the full spectrum of soil behavior types defined in the Robertson Classification, confirming that the uniformly spaced sampling strategy effectively produces a well-distributed and comprehensive CPT-based dataset suitable for machine-learning model development, training, and validation.

### Statistically distributed parameter sampling

A synthetic database was generated based on statistical distributions to represent the six CPT-derived input parameters used in the Robertson Classification framework. The *q*_*c*_ was defined over a range of 0 to 50,000 kPa and assigned a lognormal distribution, reflecting the strongly right-skewed nature of CPT tip resistance observed in natural soil deposits. Field measurements commonly show that low to moderate *q*_*c*_ values occur most frequently, while very high resistances associated with dense sands, gravels, or stiff soils are comparatively rare. This distributional behavior has been widely reported in statistical analyses of CPT data and is attributed to spatial heterogeneity, depositional processes, and stress history effects in natural soils^48^.

Similarly, *f*_*s*_ was varied between 0 and 1,000 kPa and also modeled using a lognormal distribution. The positive skewness of *f*_*s*_ ​ reflects the predominance of soils with relatively low frictional resistance, while higher values occur less frequently and are typically associated with coarse-grained or highly plastic soils. Previous CPT-based statistical studies have demonstrated that sleeve friction and related friction ratios exhibit heavy-tailed distributions due to variability in soil texture, mineralogy, and stress conditions^49^.

In contrast, *u*_*2*_, *σ*_*v*_, *σ’*_*v*_, and *u*_0_ were assigned uniform distributions within their respective ranges,0–500 kPa for *u*_*2*_​, *σ’*_*v*_ and *u*_0_, 0–1,000 kPa for *σ*_*v*_. This uniform sampling strategy was adopted to ensure that the synthetic dataset spans the full theoretical domain of stress and pore-pressure conditions relevant to CPT interpretation, without imposing site-specific hydrogeological assumptions. Such an approach is particularly suitable for synthetic data generation aimed at machine-learning model training and sensitivity analysis.

Through the multivariate combination of these CPT-derived parameters, each synthetic record was subsequently transformed into the normalized CPT parameters required by Robertson^11^ and assigned to a corresponding soil behavior type zone (Zone 1–9). Because soil behavior classification depends on nonlinear interactions among stress-normalized resistance, frictional response, and pore-pressure behavior, the resulting distribution of soil behavior zones reflects the combined effects of multiple stochastic variables rather than any single input parameter. A total of 117,649 synthetic CPT records were generated through this statistical sampling process, providing comprehensive coverage of soil behavior types defined within the Robertson Classification framework.

**Fig. 2:**
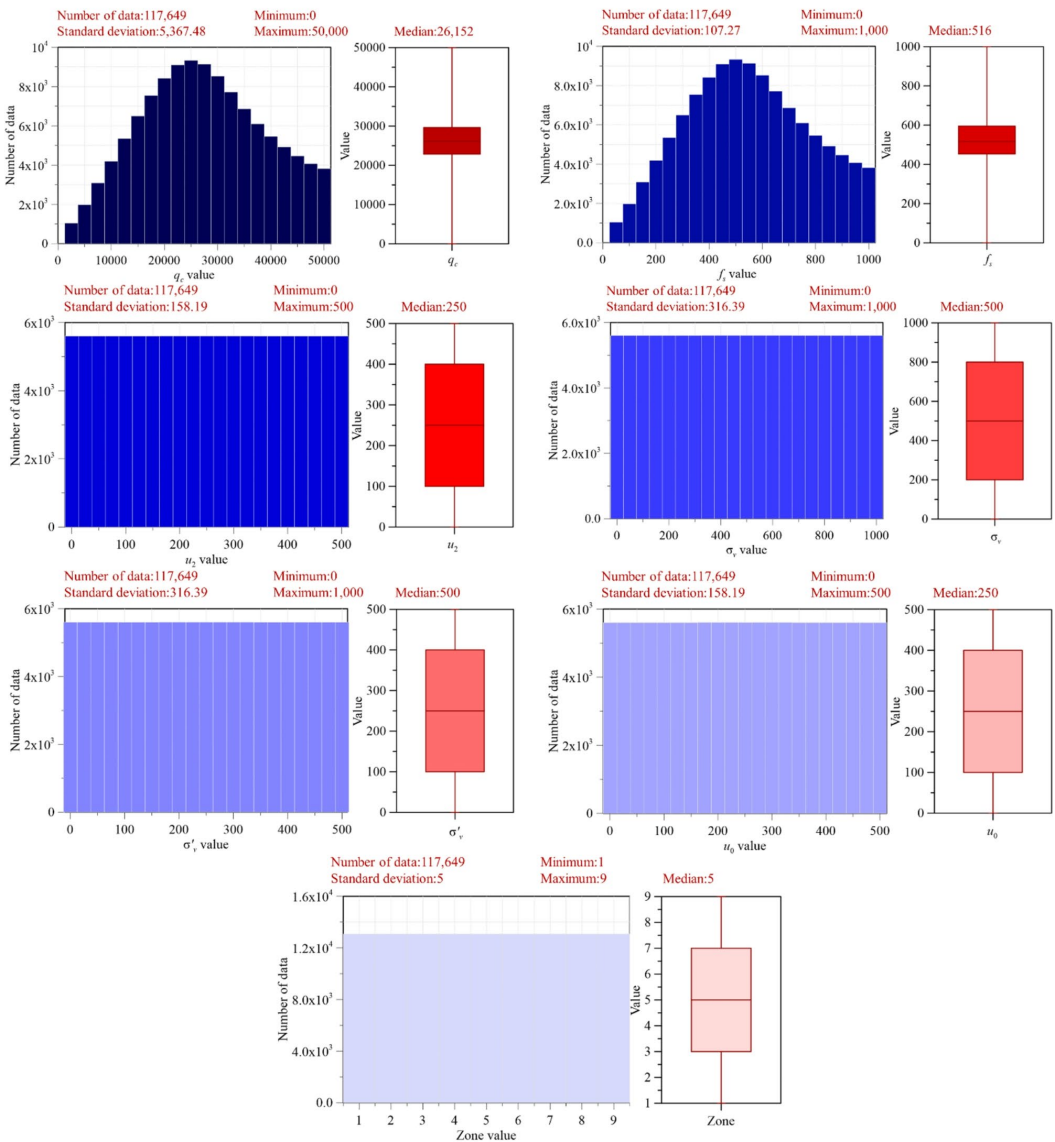
Lognormal and uniform distributions of CPT-derived parameters used in the Robertson Classification.

Figure 2 illustrates the statistical distribution patterns of the CPT-derived parameters used in this study. The parameters *q*_*c*_​ and *f*_*s*_ exhibit clear lognormal distributions characterized by positive skewness, consistent with reported field observations. In contrast, the *u*_*2*_​, *σ*_*v*_ ​, *σ’*_*v*_ ​, and *u*_0_ follow uniform distributions, reflecting the intentional design choice to evenly sample their theoretical ranges. The resulting soil behavior type zones are also uniformly distributed between Zones 1 and 9. As shown in Fig. 2, discretization of the input parameters leads to vacant intervals in the histograms, a direct consequence of interval-based sampling rather than continuous random draws. Overall, these results confirm that the synthetic dataset successfully reproduces the statistical heterogeneity, nonlinearity, and variability characteristic of CPT-based soil behavior data, supporting its suitability for subsequent machine-learning model development and validation within the Robertson Classification framework.

To visualize the variability and central tendency of the generated datasets, box plots are employed, as shown in Fig. 2. Applying this procedure to all CPT-derived parameters resulted in a synthetic dataset consisting of 117,649 unique parameter combinations. Each combination was subsequently interpreted within the Robertson Classification framework and assigned to a corresponding soil behavior type or classification zone. This categorical representation provides a robust foundation for machine-learning model development and validation, supporting stable training and reliable soil behavior classification. Furthermore, the use of statistically distributed parameter sampling enhances data diversity and reduces the risk of overfitting by exposing the models to a broad range of soil conditions, thereby improving generalization capability and predictive robustness under realistic field scenarios.

In this study, although the ranges and distributions of individual parameters were obtained from different references, the preliminary synthetic database was subsequently examined and physically unreasonable samples were removed to improve data plausibility. In addition, the relationships among total vertical stress, effective vertical stress, pore pressure, and static pore pressure were considered during the construction of the synthetic dataset. These variables were not treated as completely independent inputs, and their interdependence was taken into account to maintain physically meaningful CPT conditions.

### AI-enhanced robertson classification

This study develops four machine learning models to enhance CPT-based soil behavior classification within the Robertson Classification framework. Machine learning offers a powerful data-driven approach for capturing the complex nonlinear relationships among CPT-derived soil parameters. Four algorithms, random forest (RF), artificial neural network (ANN), support vector regression (SVR), and decision tree (DT), are employed as the primary predictive models. The analysis further involves systematic hyperparameter tuning, model validation, comparative performance assessment, and evaluation of the relative importance of input parameters.

### AI-enhanced models

Machine learning techniques provide a powerful data-driven framework for enhancing CPT-based soil behavior classification, particularly in addressing the nonlinear and multi-parameter interactions inherent in empirical systems such as the Robertson Classification. In this study, the proposed ML models were developed to improve predictive performance and reduce subjectivity in soil behavior interpretation. The selected algorithms offer complementary strengths in handling complex datasets, capturing multivariate relationships, and achieving high prediction accuracy. All models were trained using the previously described synthetic CPT dataset, allowing systematic evaluation across a wide range of subsurface conditions. Hyperparameter optimization, cross-validation, and interpretability analyses were performed to ensure robust performance and strong generalization capability.

In this study, the soil behavior type defined by Robertson^11^ was categorized into nine classes (Zone 1–9), which were encoded as ordered numerical labels and used as the output layer of the machine-learning model. The model was trained using a regression formulation to predict the corresponding SBT index. After prediction, the model outputs were compared with the original encoded labels, and the coefficient of determination and RMSE were calculated to evaluate the prediction error of the SBT index. Therefore, these statistical metrics quantify how closely the predicted SBT zone index matches the reference classification. The final engineering interpretation, however, remains a classification problem. The practical performance of the method was evaluated using classification accuracy by comparing the predicted soil types with the corresponding USCS soil classification.

The classification framework is developed based on the Robertson CPT-based soil behavior classification, and therefore its applicability follows the same general assumptions as the Robertson method. The model is intended for typical sedimentary soils commonly encountered in offshore wind farm site investigations, including sand-dominated, clay-dominated, and transitional soils interpreted from CPT measurements. The validation dataset consists of offshore CPT records, and the input parameter ranges were defined within practical geotechnical investigation conditions. Consequently, the model is suitable for CPT-based site characterization where soil behavior types can be interpreted using normalized cone resistance and friction ratio. However, the method is not intended for materials outside the scope of CPT interpretation, such as rock, very gravelly deposits, cemented soils, or highly organic soils where cone penetration resistance does not represent soil behavior reliably.

**Fig. 3:**
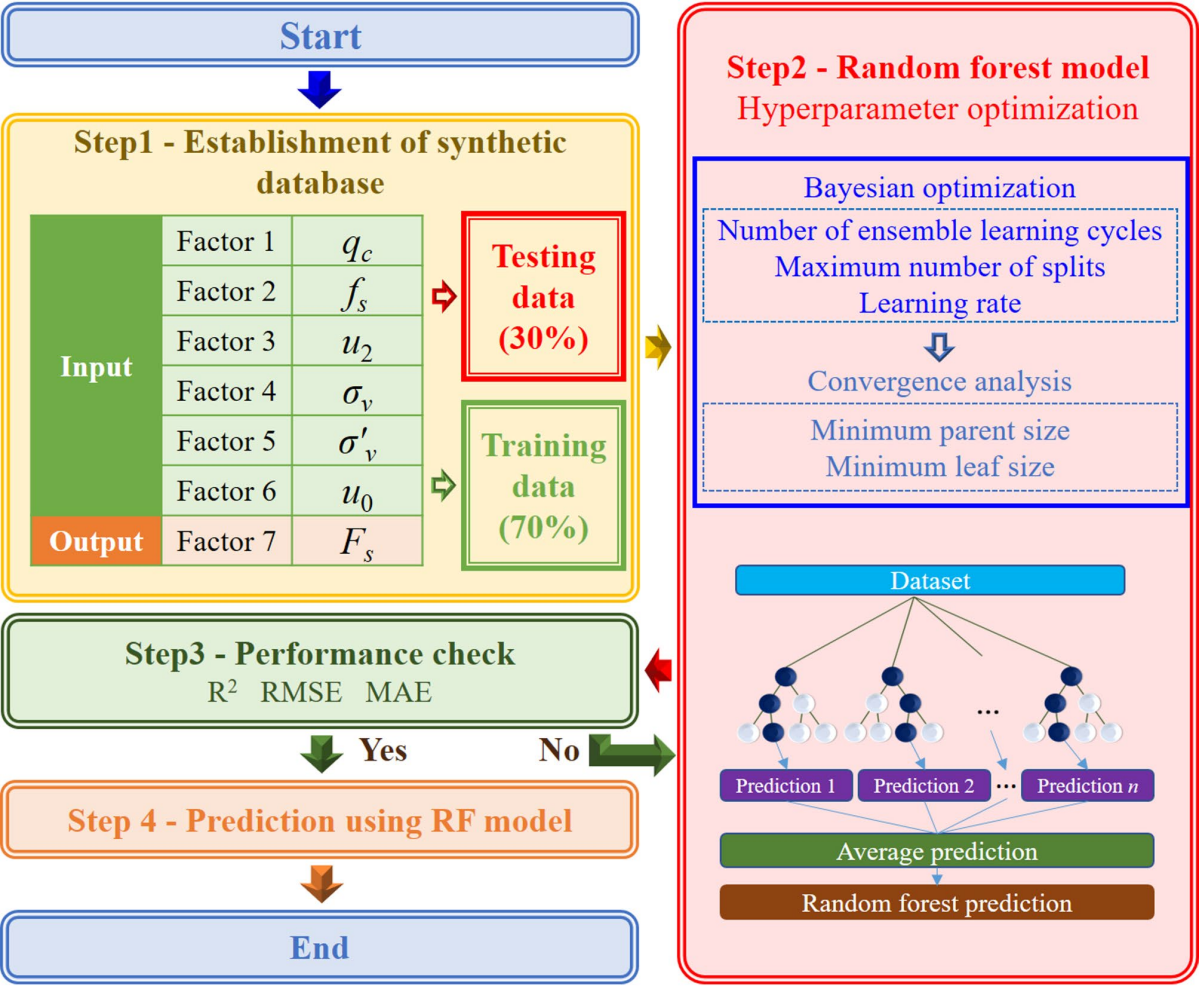
Flowchart for Robertson classification using the RF model.

This study evaluates several machine-learning models for CPT-based soil behavior classification within the Robertson Classification framework, each offering distinct strengths. RF, introduced by Breiman (2001), is an ensemble learning method that combines multiple decision trees trained on different subsets of data and features to improve generalization and reduce overfitting. Its ability to model nonlinear relationships, handle high-dimensional and heterogeneous CPT variables with minimal preprocessing, and provide built-in feature importance analysis makes RF particularly well suited for geotechnical applications, allowing quantitative assessment of the influence of parameters such as cone tip resistance, sleeve friction, and pore water pressure. Figure 3 presents the workflow of the Robertson Classification implemented using the RF model.

ANN was also employed to capture complex nonlinear interactions among CPT-derived inputs using a multilayer feedforward architecture with nonlinear activation functions. Model performance was enhanced through hyperparameter optimization, while overfitting was mitigated using dropout, early stopping, and cross-validation; although ANN typically achieve high predictive accuracy, their limited interpretability makes them more suitable when prediction performance is prioritized over transparency.

SVR was implemented using a radial basis function kernel to map CPT data into a higher-dimensional space and capture nonlinear trends through a margin-maximizing formulation. Key hyperparameters, including the regularization term, kernel width, and epsilon-insensitive loss margin, were optimized via grid search and cross-validation, resulting in a model that is robust to overfitting and outliers and well suited to the wide variability typical of CPT datasets, albeit without explicit feature importance measures.

Finally, DT models were applied due to their transparent, rule-based structure, in which CPT parameters such as pore water pressure response, sleeve friction, and tip resistance are used to recursively partition the feature space into interpretable decision pathways. DTs effectively capture nonlinear relationships and variable interactions with minimal preprocessing, making them particularly valuable for understanding how CPT parameters drive soil behavior classification outcomes within the Robertson framework, even if, like real trees, they sometimes prefer simplicity over perfection.

### Performance assessment

Model predictive performance is evaluated using three complementary regression metrics: the coefficient of determination (R^2^), root mean square error (RMSE), and mean absolute error (MAE). These measures characterize different aspects of accuracy and enable an objective comparison among the proposed machine-learning models. The corresponding formulations are provided below.

2 $${{\mathrm{R}}^{\mathrm{2}}}=1 - \frac{{\sum\nolimits_{{i=1}}^{n} {{{({y_i} - {{\hat {y}}_i})}^2}} }}{{\sum\nolimits_{{i=1}}^{n} {{{({y_i} - \bar {y})}^2}} }}$$,

where ${y_i}$ is the mean of the observed values, ${\hat {y}_i}$ is the model-predicted values, $\bar {y}$ is the average of the predicted responses, and *n* is the total number of data points. The RMSE quantifies the typical prediction error by taking the square root of the mean of the squared differences between the observed values and the corresponding model predictions, as defined in Eq. (3).

3 $${\mathrm{RMSE}}=\sqrt {\frac{{\mathrm{1}}}{n}\sum\nolimits_{{i=1}}^{n} {{{({y_i} - {{\hat {y}}_i})}^2}} }$$

MAE characterizes the typical prediction error by taking the mean of the absolute deviations between the observed responses and the corresponding model estimates, as defined in Eq. (4)

4 $${\mathrm{MAE}}=\frac{{\mathrm{1}}}{n}\sum\nolimits_{{i=1}}^{n} {\left| {{y_i} - \hat {y}} \right|}$$

The MAE represents the average magnitude of the absolute residuals between the predicted and observed values, providing a direct measure of prediction accuracy. In the subsequent validation stage, this metric is used, together with the other evaluation indices, to quantify predictive performance and assess the overall reliability of the developed models. It is important to note that classification accuracy serves as the primary metric for evaluating model performance, whereas R² and RMSE are retained as supplementary indicators to describe the numerical consistency of the predictions. Although no additional classification-based analyses have been introduced, the roles and applicability of these metrics have been explicitly clarified to avoid potential misunderstanding. In addition, the implications of misclassification between adjacent and distant SBT zones are discussed to better reflect the categorical and ordered nature of the problem.

### Validation of AI-enhanced models

Model validation in this study is carried out through four complementary analyses: hyperparameter optimization, evaluation of overall predictive performance, assessment of input parameter importance, and investigation of model robustness under missing-data scenarios. Together, these procedures ensure that the RF, ANN, SVR, and DT models are rigorously tested for accuracy, stability, and generalization when applied to CPT-based soil behavior classification within the Robertson Classification framework.

### Hyperparameter optimization

**Fig. 4:**
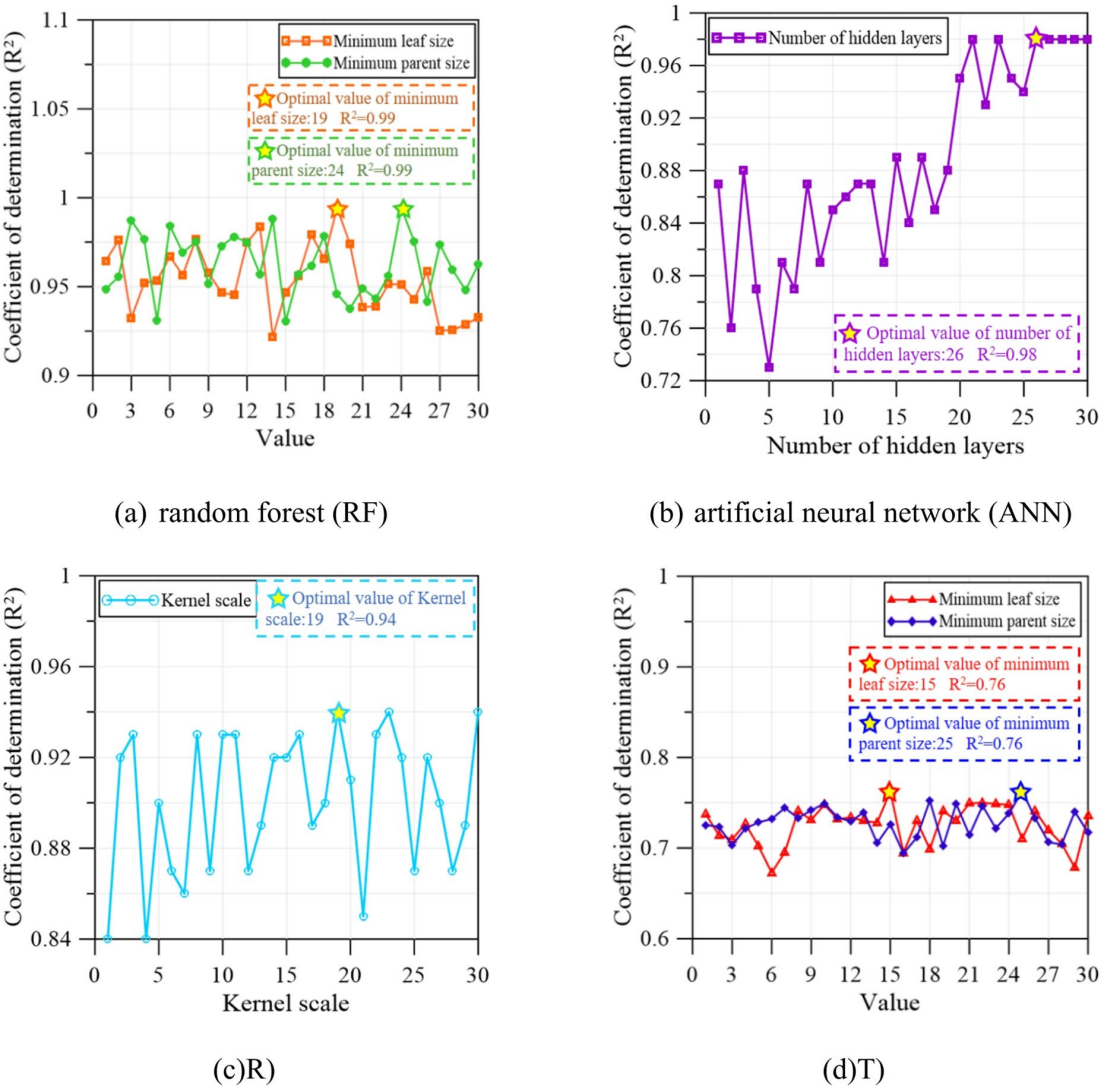
Results of the hyperparameter optimization using AI-enhanced models. (c) support vector regression, (SVR), (d) decision tree, (DT).

Hyperparameter optimization was carried out for the four AI models including RF, ANN, SVR, and DT to enhance prediction accuracy and model stability. The outcomes of the optimization process are summarized in Fig. 4. For the RF model, the influence of minimum parent size and minimum leaf size on model performance was systematically evaluated, and the optimal configuration was identified at a leaf size of 19 and a parent size of 24, corresponding to a coefficient of determination (R²) of 0.99, as shown in Fig. 4**(a)**. In the ANN model, the network architecture was systematically optimized by varying the number of hidden layers, and the best predictive performance was obtained with an R^2^ value of 0.98 when 26 hidden layers were used, as shown in Fig. 4**(b)**. For the SVR model, the kernel scale parameter was tuned, and optimal performance was achieved at a kernel scale of 19, corresponding to an R^2^ of 0.94 (Fig. 4**(c)**). In the DT model, hyperparameter optimization was conducted by adjusting the leaf size and parent size, with the highest predictive accuracy obtained at a leaf size of 15 and a parent size of 25, yielding an R^2^ of 0.76 (Fig. 4**(d)**). Overall, these results demonstrate that careful hyperparameter optimization can substantially improve model performance, highlighting the effectiveness of AI-enhanced modeling strategies for CPT-based soil behavior prediction within the Robertson Classification framework.

A hyperparameter sensitivity analysis was conducted for the RF, ANN, SVR, and DT models using the coefficient of determination (R²).For the Random Forest model, R² ranged from 0.90 to 1.00 across minimum leaf and parent node sizes, indicating low sensitivity; the optimal parameters were minimum leaf size = 19 and minimum parent size = 24.For the Artificial Neural Network model, R² increased with hidden layers and converged at 26, which was therefore adopted.For the Support Vector Regression model, R² varied from 0.84 to 0.94 across kernel scales; the optimal kernel scale was 19.For the Decision Tree model, R² ranged from 0.66 to 0.76 across minimum leaf and parent node sizes, with optimal values of minimum leaf size = 15 and minimum parent size = 25.Overall, performance remained stable over reasonable hyperparameter ranges, and the selected settings represent robust, best-performing configurations.

### Overall performance of the ML models

The predictive performance of the AI-enhanced models, including RF, ANN, SVR, and DT, was systematically evaluated using statistical indicators such as the R², RMSE, and MAE, providing a comprehensive assessment of regression accuracy and model reliability based on the synthetic CPT dataset. These metrics were used to compare differences in predictive capability among the models. As summarized in Table 2, the RF model exhibited the best overall performance, achieving an R² of 0.99, an RMSE of 1.47 × 10^− 2,^ and an MAE of 9.65 × 10^− 3^. The ANN model showed similarly strong performance, with an R² of 0.98, an RMSE of 2.64 × 10^− 2^, and an MAE of 1.02 × 10^− 2^, whereas the SVR model demonstrated moderate accuracy (R² = 0.94, RMSE = 8.73 × 10^− 2^, MAE = 5.51 × 10^− 2^). In contrast, the DT model yielded the lowest predictive performance, with an R² of 0.76, an RMSE of 1.42 × 10^− 1^, and an MAE of 8.14 × 10^− 2^. These results indicate that RF consistently outperforms the other models, likely due to its ensemble-based structure, which effectively captures nonlinear interactions among CPT-derived parameters while mitigating overfitting. Consequently, RF is identified as the most robust and reliable algorithm for AI-enhanced CPT-based soil behavior classification within the Robertson Classification framework.

**Fig. 5:**
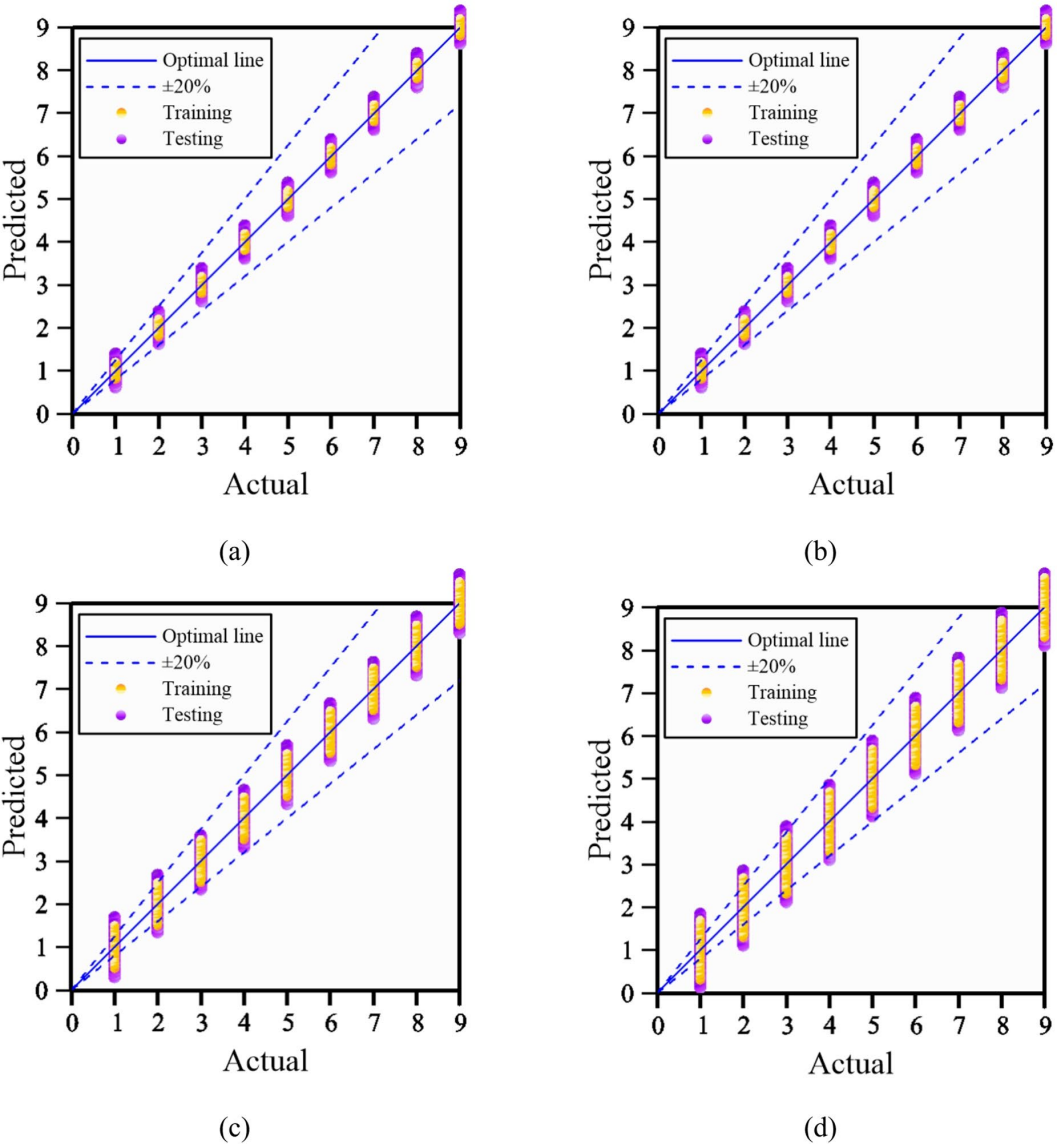
Performance of ML models using optimization. (**a**) RF model with Bayesian optimization, (**b**) ANN model with optimization, (**c**) SVR model with Bayesian optimization, (**d**) DT model with optimization.

Figure 5 presents the performance of the machine-learning models following hyperparameter optimization. The results indicate that the RF model achieved the highest predictive accuracy, with an R² value of 0.99, followed closely by the ANN model with an R² of 0.98, and both models show strong agreement between predicted and observed values. In comparison, the SVR model (R² = 0.94) and the DT model (R² = 0.76) exhibit larger discrepancies between predictions and measurements, reflecting relatively lower predictive capability. Figure 5 further presents the outcomes of Bayesian optimization, demonstrating that the optimized models produce predictions that are highly consistent with the observed reference values. Based on these results, the RF model was subsequently coupled with Bayesian optimization to further refine its hyperparameters and to evaluate predictive performance using both error-based metrics and goodness-of-fit indicators.

**Table 2:** Comparison of performance assessment on the test data for different ML algorithms.

*R* ^2^	RF	ANN	SVR	DT
	0.99	0.98	0.94	0.76
RMSE	1.47 × 10^− 2^	2.64 × 10^− 2^	8.73 × 10^− 2^	1.42 × 10^− 1^
MAE	9.65 × 10^− 3^	1.02 × 10^− 2^	5.51 × 10^− 2^	8.14 × 10^− 2^

### Parameter importance

**Fig. 6:**
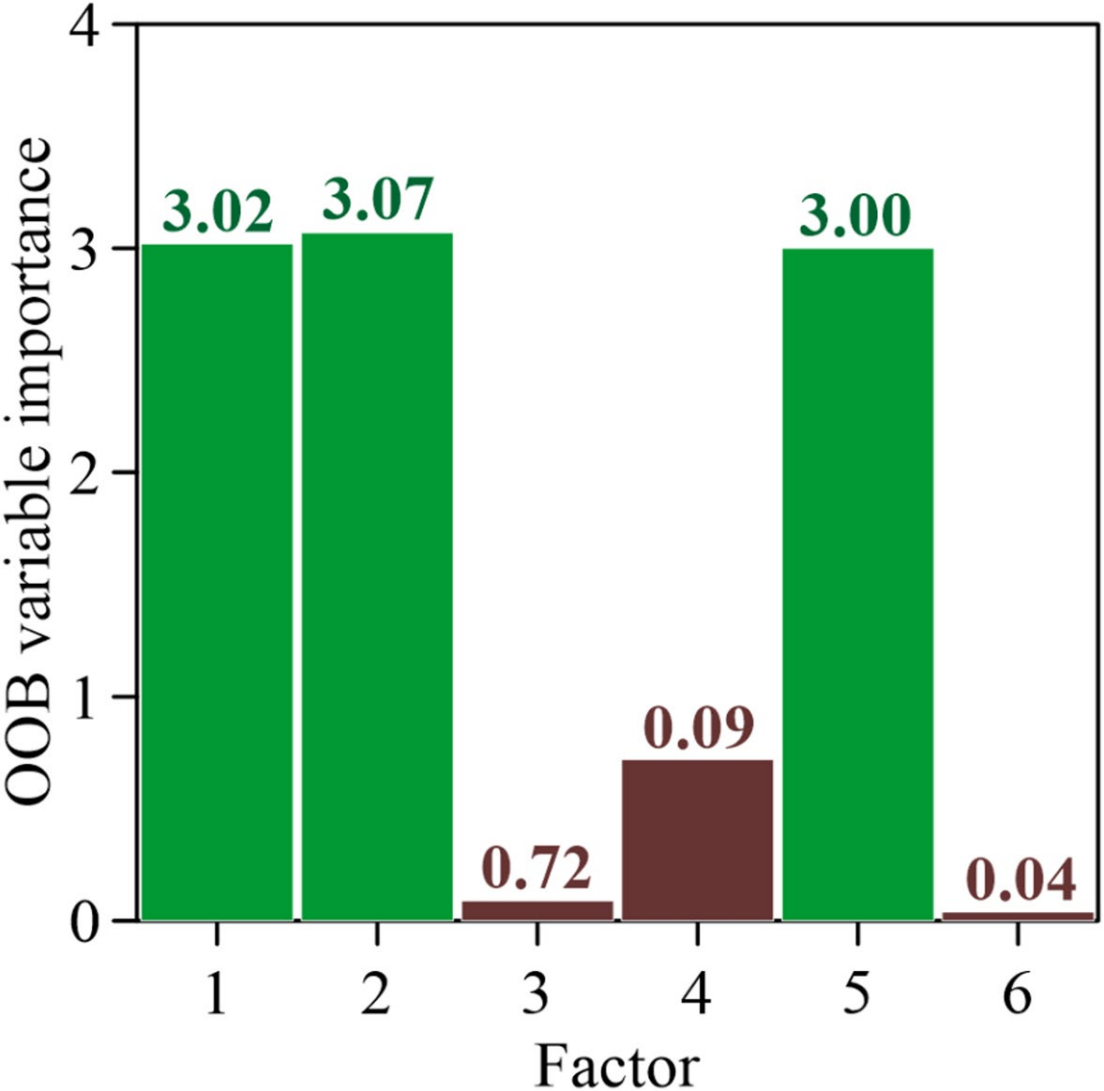
Importance for the factors.

Parameter importance was assessed using the out-of-bag (OOB) citation estimates obtained from the RF model, providing an objective measure of the relative contribution of each CPT-derived input parameter to the prediction of soil behavior within the Robertson Classification framework. As illustrated by the analysis results in Fig. 6, the six factors considered, *q*_*c*_, *f*_*s*_, *u*_2_, σ_*v*_, σ*’*_*v*_, and *u*_0_, exhibit markedly different levels of influence on the model output. Factor 1 is tip resistance, factor 2 is sleeve friction, factor 3 is pore water pressure, factor 4 is total vertical stress, factor 5 is effective vertical stress, factor 6 is static pore water pressure, and factor 7 is the soil behavior type zone. As shown in Fig. 6, the OOB importance values for *q*_*c*_, *f*_*s*_, *u*_2_, σ_*v*_, σ*’*_*v*_, and *u*_0_ are 3.02, 3.07, 0.09, 0.72, 3.00, and 0.04, respectively. Among these variables, σ*’*_*v*_ and *q*_*c*_ emerge as the most influential factors, highlighting the dominant role of stress conditions and soil strength in governing CPT-based soil behavior interpretation. *f*_*s*_ also shows relatively high importance, indicating its significance in distinguishing between clay-like and sand-like responses. In contrast, pore water pressure–related variables, particularly *u*_0_, contribute less to the overall prediction, suggesting a secondary influence under the analyzed conditions. Overall, the OOB importance analysis demonstrates that mechanical response and stress-related parameters are the primary drivers controlling soil behavior classification in the Robertson Classification system, consistent with the behavior-based nature of CPT interpretation.

In the Robertson framework, soil behavior type is primarily determined using normalized cone resistance and friction ratio. The effective vertical stress is required for stress normalization of cone resistance, and therefore directly controls the interpretation of soil behavior zones. The cone tip resistance reflects soil strength and stiffness and forms the principal indicator distinguishing dense granular soils from soft cohesive soils. The sleeve friction, together with cone tip resistance, defines the friction ratio, which is the key index used to differentiate sand-like and clay-like behavior and is strongly related to soil plasticity. Therefore, the parameters identified as important by the machine learning model correspond directly to the fundamental variables used in the Robertson classification theory. The agreement between the OOB importance ranking and geotechnical principles indicates that the model captures physically meaningful relationships rather than spurious statistical correlations.

Additionally, the relatively low importance of pore pressure is expected because it is not directly involved in the Robertson chart formulation. Alternative CPTu-based interpretation frameworks^50^, which explicitly incorporate pore pressure behavior, could lead to different importance rankings. The purpose of the feature-importance analysis in this study is therefore not to identify new soil behavior mechanisms, but to verify that the model captures the physical relationships embedded in the chosen classification framework. This also represents a limitation: the importance ranking is dependent on the selected soil classification method.

### Effect of parameter sampling on prediction accuracy

**Fig. 7:**
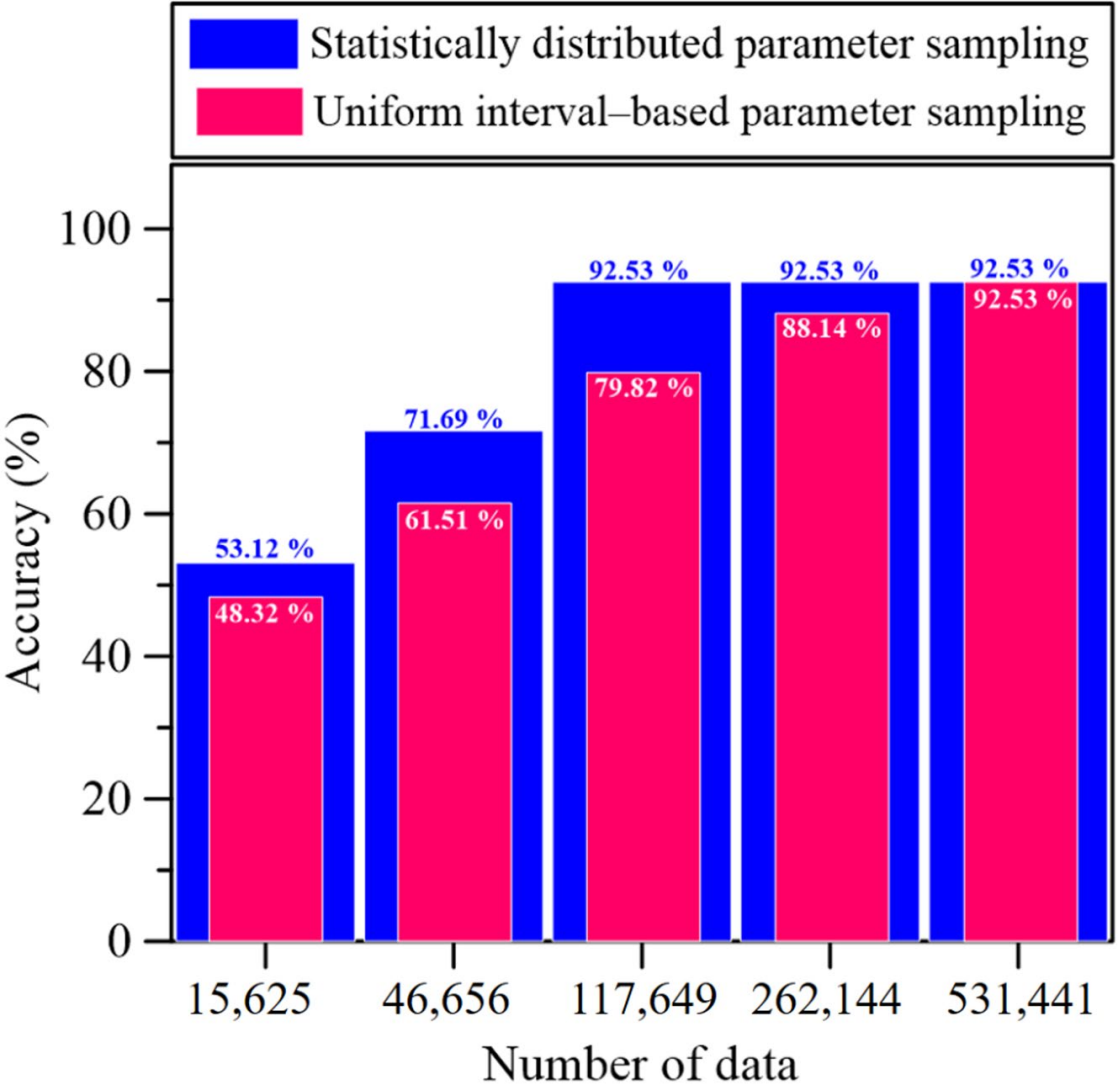
Prediction accuracy under different parameter interval.

To investigate the influence of sampling resolution on model performance, a series of synthetic CPT datasets were generated using different parameter intervals ranging from 5 to 9, producing database sizes between 15,625 and 531,441 samples. As summarized in Table 3, a clear improvement in model performance was observed with increasing data volume. When the dataset comprised 15,625 samples (interval = 5), the predictive capability was relatively limited (R² = 0.79). Increasing the interval to 6 and 7, corresponding to 46,656 and 117,649 samples, led to substantial performance gains, with R² values of 0.87 and 0.92, respectively. These results suggest that model reliability improves significantly once the dataset size exceeds approximately 117,649 samples, highlighting the strong dependence of CPT-based soil behavior prediction on data availability within the Robertson Classification framework. Figure 7 further illustrates the variation in prediction accuracy with respect to parameter intervals. For each interval setting, synthetic CPT datasets were generated and corresponding RF models were trained. Model accuracy was then evaluated using 229,808 offshore wind farm CPT records. The results indicate that, once the synthetic dataset exceeded 229,808 samples, the model attained a prediction accuracy of 92.53%. It is found that finer sampling resolution and the resulting larger data volume substantially enhance the robustness and reliability of soil behavior classification based on the Robertson Classification.

Both uniform interval based parameter sampling and statistically distributed parameter sampling were used to train the model, and their predictive accuracies were compared. The results indicate that the statistically distributed parameter sampling provides better predictive performance. Therefore, the dataset generated in statistically distributed parameter sampling was adopted as the final training dataset, as shown in Fig. 7.

**Table 3:** Accuracy under varying parameter sampling resolutions.

Number of data	Error metrics		
	*R* ^2^	RMSE	MAE
15,625	0.79	8.31 × 10^− 2^	6.11 × 10^− 2^
46,656	0.87	7.62 × 10^− 2^	3.54 × 10^− 2^
117,649	0.99	1.49 × 10^− 2^	9.83 × 10^− 3^
262,144	0.99	1.47 × 10^− 2^	9.81 × 10^− 3^
531,441	0.99	1.42 × 10^− 2^	9.75 × 10^− 3^

### Offshore wind farm cases prediction using the ai-enhanced model

**Fig. 8:**
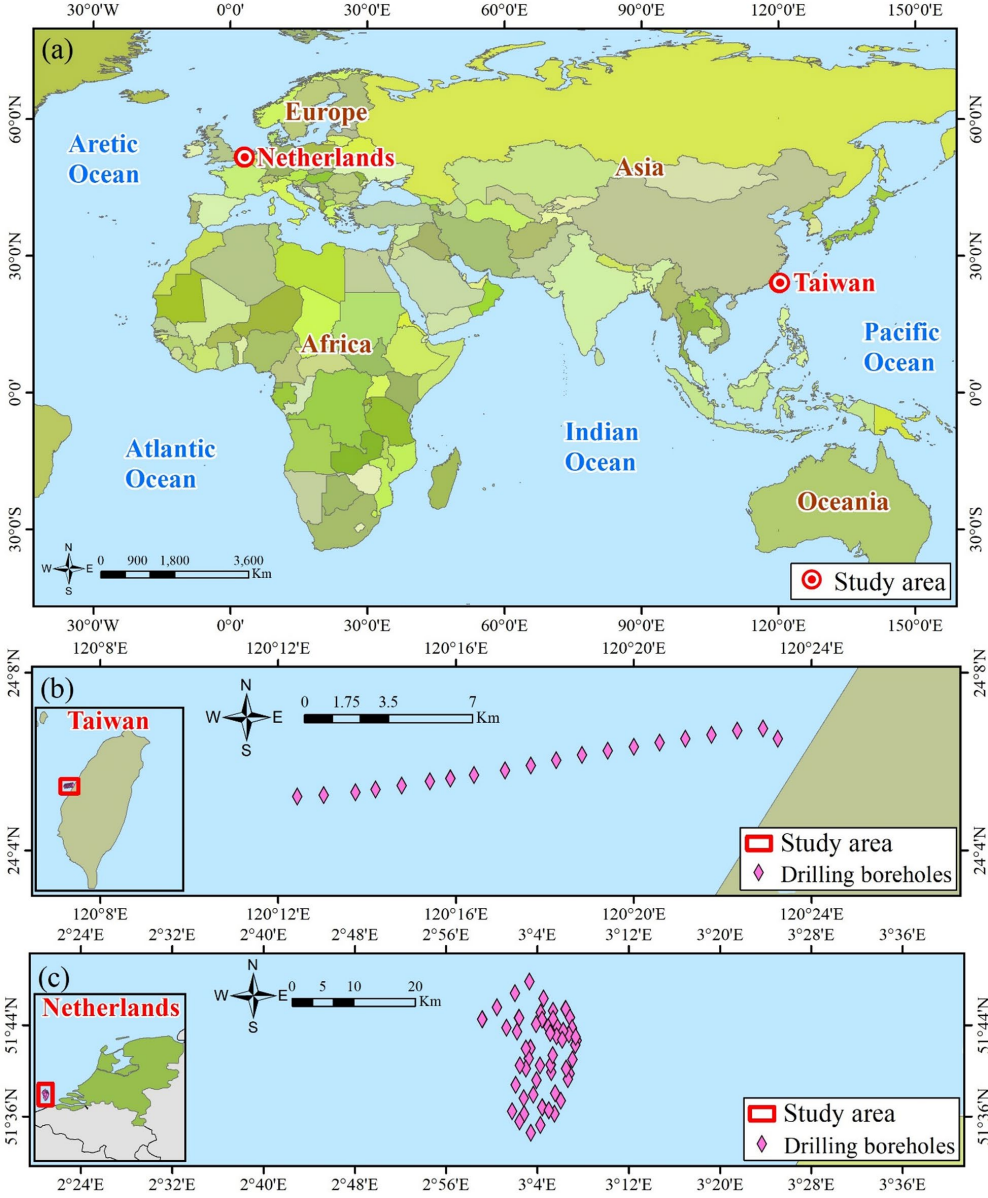
Geographical distribution of actual soil samples (this figure was created with ArcGIS 10.8 software, obtained from https://www.arcgis.com/index.html).

To evaluate the practical applicability of the proposed AI-enhanced framework, an external validation was performed using CPT-based soil investigation data collected from offshore wind farm sites, as illustrated by the validation cases compiled in Fig. 8. A total of 229,808 CPT records obtained from 99 drilling boreholes were assembled, comprising 13,118 records from 24 boreholes in Taiwan and 216,690 records from 75 boreholes in the Netherlands. These datasets encompass a broad range of soil behavior types defined within the Robertson Classification framework, providing a comprehensive basis for model validation under realistic offshore field conditions. For each CPT record, the soil behavior type or corresponding classification zone was independently interpreted following established Robertson Classification criteria, allowing direct comparison with the AI-based predictions. The close agreement between the predicted results and the reference classifications derived from field CPT data demonstrates the robustness, reliability, and practical applicability of the proposed method for CPT-based soil behavior classification in offshore wind farm geotechnical investigations.

In this study, the impact of such discrepancy was indirectly evaluated through external validation using real offshore CPT records. The model was trained exclusively on the synthetic dataset and then applied to 229,808 measured CPT records from offshore wind farm sites. Despite the potential distribution differences, the model achieved a prediction accuracy of approximately 92.53%. If a significant mismatch existed between the synthetic and measured data domains, a substantial degradation of predictive performance would be expected. The strong agreement between predicted and reference classifications therefore indicates that the synthetic dataset successfully captures the essential soil behavior space rather than merely reproducing specific numerical distributions. The purpose of the synthetic dataset is to represent the theoretical range of CPT responses within the Robertson classification framework, allowing the model to learn general soil behavior relationships. The external validation against measured CPT data provides the practical assessment of model performance under real engineering conditions.

### Validation using offshore wind farm cases

Table 4 summarizes the offshore wind farm CPT dataset used for external validation in this study. All 229,808 CPT records obtained from 99 drilling boreholes were reserved exclusively for the prediction of the RF and were not used in model training or internal testing. The RF-based predictions were evaluated against independent reference classifications to assess predictive performance. Each CPT record includes CPT-derived input parameters and a corresponding soil behavior classification determined using established criteria. To quantify classification accuracy, soil behavior types predicted within the Robertson Classification framework were compared with soil groups independently classified according to the Unified Soil Classification System (USCS). Although the Robertson Classification and USCS represent different classification philosophies, previous studies have demonstrated consistent correspondence between CPT-based soil behavior types and USCS groups, making such cross-comparison a practical validation approach in offshore engineering. The proposed RF model achieved a prediction accuracy of 92.53%, demonstrating strong agreement with USCS-based classifications and confirming the robustness and reliability of the AI-enhanced framework.

**Table 4:** Dataset of 229,808 actual soil samples.

Location	Number of drilling boreholes	Zone	Number of data	Source
Taiwan	24	1	659	Phase II Offshore Wind Power Project, 2021
		2	5,227	
		3	2,472	
		4	4,397	
		5	363	
Netherlands	75	2	90	Netherlands Enterprise Agency^51^,
		4	48,422	
		5	53,862	
		6	83,646	
		7	30,670	

### Predictions with missing input parameters

In practical geotechnical applications, CPT field data may be incomplete or partially unavailable, leading to missing input variables during CPT-based soil classification. To examine the robustness of the proposed AI-enhanced models under such conditions, this study evaluates their predictive performance when one of the CPT-derived input parameters used in the Robertson Classification framework is absent. Missing inputs were replaced with randomly generated values to represent the uncertainty commonly encountered in real-world CPT datasets. The resulting effects on prediction accuracy are summarized in Table 5. In this study, multiple random realizations were introduced and the model was repeatedly executed, and the reported prediction accuracy represents the average accuracy from these runs. This procedure corresponds to a Monte Carlo type robustness evaluation, which minimizes the influence of any single random value and reflects the overall tolerance of the model to missing CPT measurements. The purpose of the analysis is to assess model robustness under information deficiency rather than to simulate the actual missing-data pattern of a particular site. Compared with probabilistic CPT interpretation methods (e.g^25,54^.,), which quantify classification uncertainty through statistical modeling of measurement variability, the proposed framework provides a deterministic soil classification when some parameters are unavailable. The advantage of the present method is that it can still produce a usable classification result without requiring prior probabilistic characterization, while its limitation is that it does not directly quantify classification uncertainty.

When all CPT parameters were available, the model achieved a prediction accuracy of 92.53%, providing a reference baseline for assessing performance degradation under incomplete data scenarios. As summarized in Table 5, the predictive performance of the model decreases when individual CPT-derived input parameters used in the Robertson Classification framework are missing^55^^[,56^^[,57^.

Among the missing-parameter scenarios, the most pronounced reduction in accuracy occurred when *q*_*c*_ or *f*_*s*_ was excluded, with accuracies dropping to 58.74% and 55.31%, respectively, highlighting their fundamental role in CPT-based soil behavior interpretation. A substantial decrease was also observed when σ*’*_*v*_ was omitted, resulting in an accuracy of 60.42%, emphasizing the importance of stress-related variables in normalizing CPT responses^58,]^^59^.

In contrast, the absence of *u*_2_ and *u*_0_ led to only minor performance degradation, with accuracies of 91.04% and 91.69%, respectively. Excluding σ_*v*_ produced a moderate reduction in accuracy to 84.36%. These results indicate that the proposed ML framework demonstrates reasonable robustness to incomplete CPT data; however, parameters directly associated with soil strength, frictional response, and effective stress are critical for maintaining reliable soil behavior classification within the Robertson Classification system.

This study also compares the proposed RF-based model with two CPT-based machine-learning soil classification methods reported by Reale et al.^52^, and Kurup and Griffin^53^. As shown in Table 5, their reported prediction accuracies of 89.47% and 86.00%, respectively, are lower than the 92.53% achieved by the proposed model, demonstrating its superior predictive performance and effectiveness for CPT-based soil behavior classification within the Robertson Classification framework.

To assess the reliability of predictions under conditions of missing input parameters, unknown variables were replaced with randomly generated values, and the trained model was applied to simulate 229,808 offshore wind farm CPT records. Because the randomly generated inputs differ for each simulation, prediction accuracy was not derived from a single run. Instead, the reported accuracy represents the average value obtained from multiple repeated model runs, with the required number of runs determined through a convergence analysis.

**Fig. 9:**
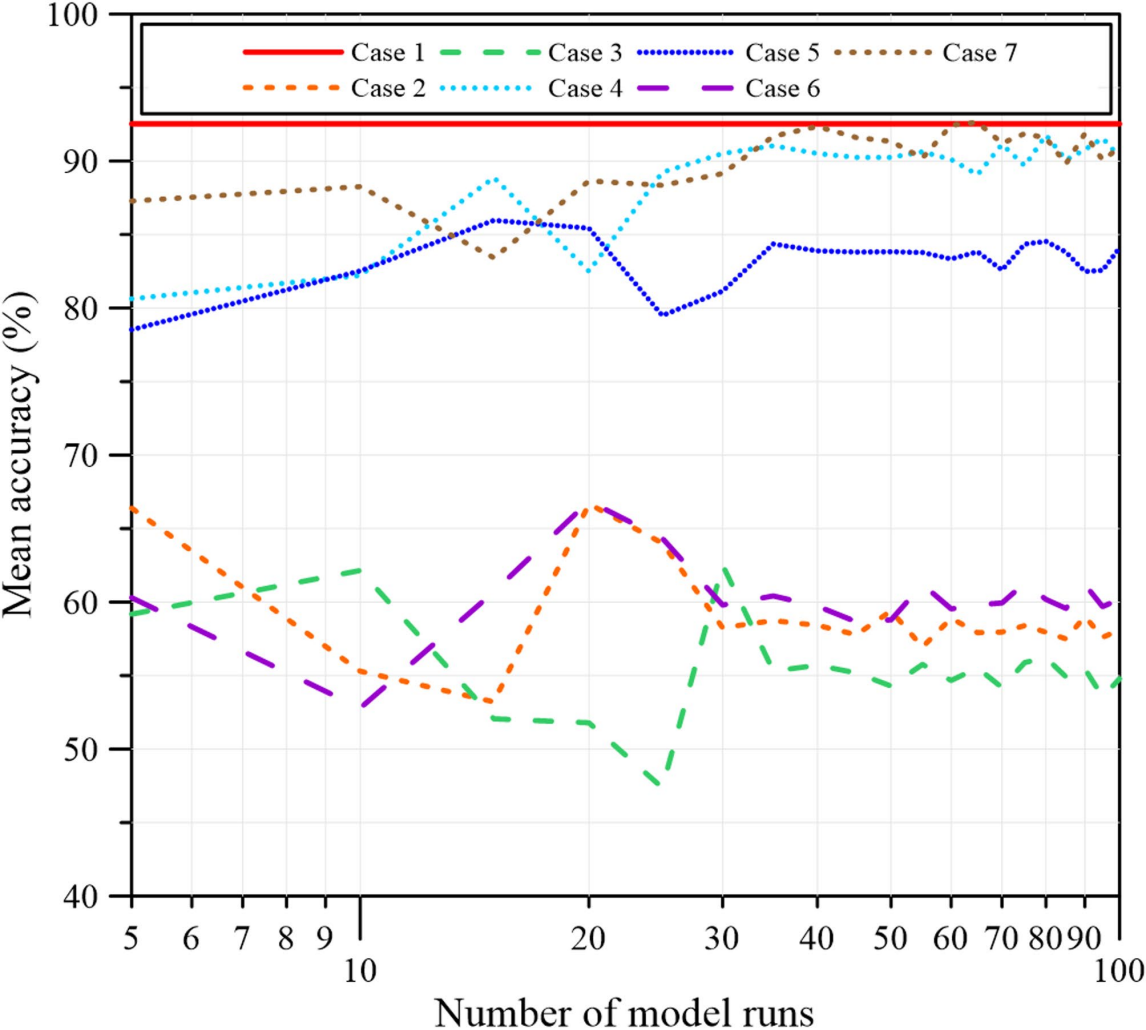
Convergence of mean accuracy for 100 model runs.

As shown in Fig. 9, prediction accuracy varies with the number of runs and gradually stabilizes for 100 model runs. In addition to mean accuracy, the standard deviation was calculated to assess the variability and consistency of the repeated predictions, providing a quantitative measure of run-to-run uncertainty. The standard deviation was calculated using Eq. (5).

5 $$\sigma =\sqrt {\frac{{\sum\limits_{{i={\mathrm{1}}}}^{n} {{{\left( {{x_i} - \overline {x} } \right)}^2}} }}{{n - 1}}}$$

where $\sigma$ represents the standard deviation, *n* is the total number of simulation runs, ${x_i}$ the accuracy of each run, and $\overline {x}$ represents the mean of all simulation outcomes.

**Fig. 10:**
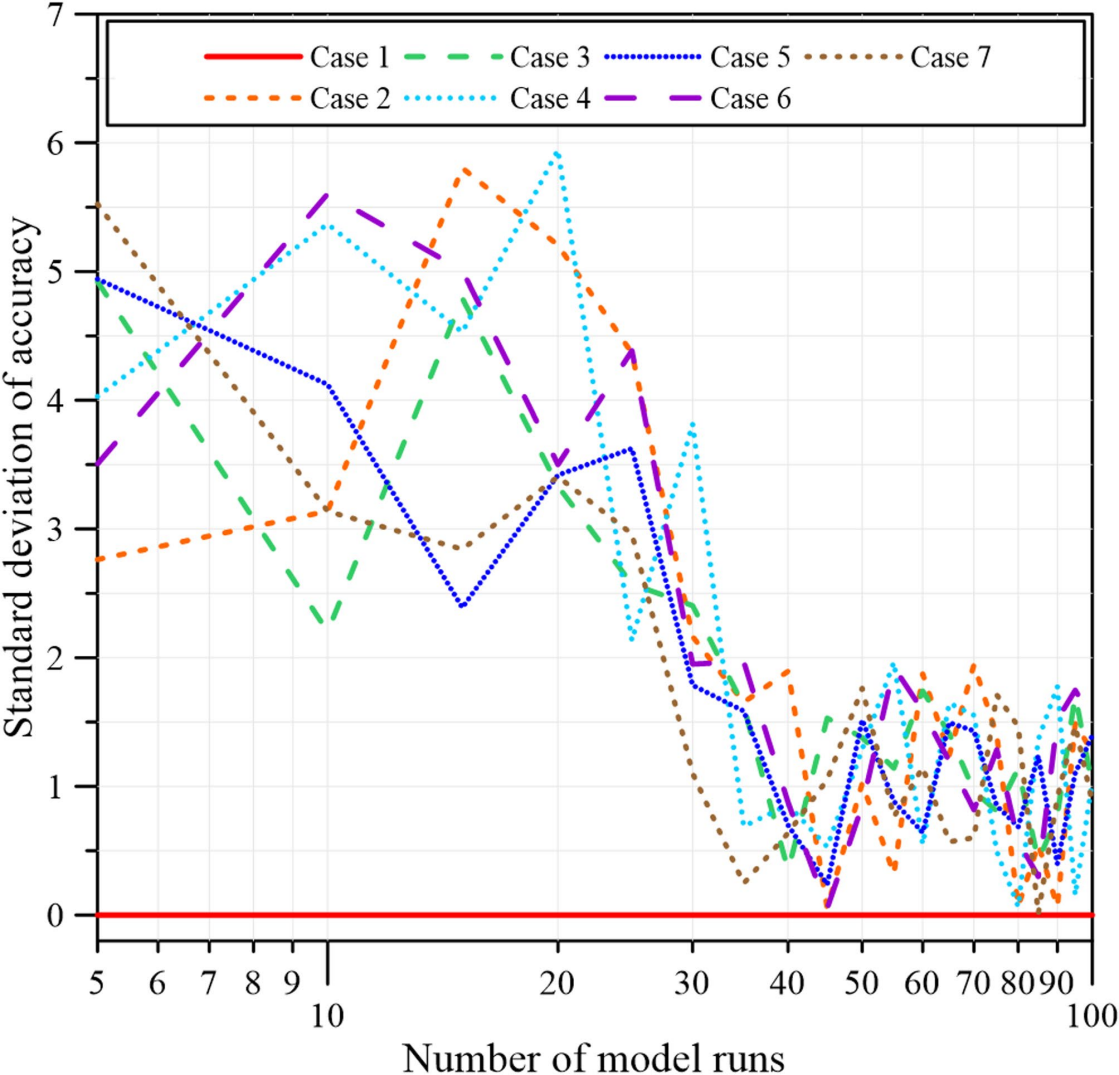
Convergence of standard deviation of accuracy for 100 model runs.

Figure 10 shows the evolution of the standard deviation with increasing numbers of model runs. As the number of simulations increases, the standard deviation gradually decreases and stabilizes for 100 model runs, indicating that the model predictions have converged and confirming the reliability of using averaged results for the final performance evaluation.

Moreover, the value of 92.53% listed for “^11^” in Table 5 does not represent a referenced value from the literature. Instead, it corresponds to the classification accuracy obtained in this study. Specifically, the same set of 229,808 CPT records was independently classified using different methods, and the predicted soil types were compared with soil groups determined according to the USCS classification. Therefore, the reported percentage represents the prediction accuracy of each method evaluated on the same dataset. The Robertson method was evaluated in the same manner as the proposed model, and its accuracy reflects its performance on the dataset rather than a literature value.

The discrepancies between predicted results and the reference interpretation are mainly concentrated in the transitional zones of the zones 3–6, particularly zone 5. This behavior is expected because the USCS classification is based on soil composition, whereas the Robertson classification reflects penetration response and drainage behavior during shearing. Accordingly, soils containing fines or exhibiting low permeability may be classified as sand in USCS but behave as silt-like materials during CPT penetration, leading to classification differences in transitional zones.

**Table 5:** Comparison of prediction accuracy across different models.

scenarios	Description	Accuracy (%)
1	All parameter included	92.53
2	Missing *q*_*c*_	58.74
3	Missing *f*_*s*_	55.31
4	Missing *u*_2_	91.04
5	Missing *σ*_*v*_	84.36
6	Missing *σ’*_*v*_	60.42
7	Missing *u*_0_	91.69
8	Robertson^11^,	92.53
9	Reale et al.^52^,	89.47
10	Kurup and Griffin^53^,	86

Table 6 illustrates the prediction accuracy of the four machine-learning models evaluated using the offshore wind farm CPT dataset described earlier. The trained models were applied to 229,808 CPT records obtained from 99 drilling boreholes, and prediction accuracy was quantified by comparing the soil behavior classifications predicted within the Robertson Classification framework with the corresponding soil groups independently determined using the USCS. This comparison provides an objective measure of model performance under realistic field conditions and enables assessment of the consistency between CPT-based AI predictions and conventional soil classification results.

The classification results based on CPT-derived data within the Robertson Classification framework reveal distinct differences in predictive performance among the evaluated machine-learning models, as illustrated in Table 6. The RF model achieved the highest classification accuracy of 92.53%, indicating the most reliable performance in predicting soil behavior types from CPT inputs. The ANN model followed closely with an accuracy of 90.18%, demonstrating strong predictive capability and effective learning of nonlinear relationships. The SVR model attained a moderate accuracy of 85.67%, reflecting reasonable but comparatively lower classification performance. In contrast, the DT model exhibited the lowest accuracy of 75.42%, suggesting limited generalization capability relative to the ensemble and kernel-based approaches. Overall, these findings demonstrate that the RF model exhibits superior robustness and predictive reliability for CPT-based soil behavior classification within the Robertson Classification framework.

Additional machine-learning algorithms, Logistic Regression (LR) and Extreme Gradient Boosting (XGBoost), were implemented using the same dataset and evaluation procedure for comparison. The results are summarized in Table 6. The prediction accuracy of LR is 91.82%, and XGBoost achieves an accuracy of 90.36%. The relatively lower performance of LR is associated with its linear decision boundary assumption, which limits its ability to capture nonlinear interactions among CPT parameters, while XGBoost shows greater sensitivity to data variability and measurement noise. In contrast, the ensemble averaging mechanism of RF provides improved robustness when handling heterogeneous geotechnical data, and RF achieves the highest prediction accuracy of 92.53%.

**Table 6:** Prediction accuracy for four ML models.

scenarios	Machine learning	Accuracy (%)
1	Random forest (RF)	92.53
2	Artificial neural network (ANN)	90.18
3	Support vector regression (SVR)	85.67
4	Decision tree (DT)	75.42
5	Logistic regression (LR)	91.82
6	Extreme Gradient Boosting (XGBoost)	90.36

### Prediction uncertainty using monte carlo simulation

Prediction uncertainty for the test dataset was evaluated using a Monte Carlo simulation approach. A total of 229,808 offshore wind farm CPT records were used to compare the soil behavior classifications derived from field data with those predicted by the proposed model within the Robertson Classification framework. For each prediction, the associated uncertainty range was estimated based on repeated simulations. The results indicate that all model-predicted soil behavior indices fall within the 95% confidence interval of the reference values, demonstrating the stability and reliability of the proposed predictive framework. Moreover, the consistently narrow confidence intervals suggest low prediction uncertainty and strong robustness of the model, confirming the effectiveness of the integrated machine-learning approach for CPT-based soil behavior classification^60^^[,61^.

## Conclusion

This study proposes an AI-enhanced framework for CPT-based soil behavior classification within the Robertson Classification system. A comprehensive synthetic dataset was constructed using six key CPT-derived input parameters, including cone tip resistance, sleeve friction, pore water pressure response, total stress, effective stress, and static pore water pressure. These data were used to train and evaluate multiple ML models, and the conclusions are as follows:

1. An AI-enhanced soil behavior classification framework based on the Robertson Classification was successfully developed using RF, ANN, SVR, and DT models. Comparative evaluation demonstrated that the RF model consistently outperformed the other algorithms in terms of predictive accuracy, robustness, and generalization capability. Feature importance analysis based on OOB estimates further revealed that stress-related parameters and cone resistance play dominant roles in governing CPT-based soil behavior classification.
2. The sampling resolution of the synthetic dataset was found to have a significant influence on model performance. As the dataset size increased through finer parameter discretization and statistically distributed sampling, predictive accuracy improved substantially. Once the synthetic dataset exceeded approximately 229,808 samples, the RF model achieved a stable prediction accuracy of 92.53% when validated against offshore wind farm CPT data, demonstrating that larger and more diverse datasets markedly enhance model reliability and stability.
3. The proposed AI models exhibited robust and accurate performance under realistic engineering conditions. Validation using 229,808 CPT records from 99 offshore wind farm boreholes showed strong agreement with USCS soil classifications. Although the RF model remained stable with missing inputs, the absence of key parameters reduced accuracy, emphasizing their importance. Overall, the framework provides a reliable and practical tool for CPT-based soil behavior classification in offshore geotechnical applications.
4. The proposed machine-learning framework is not intended to replace the Robertson chart. Instead, it addresses a different practical problem: soil behavior classification when CPT measurements are incomplete, partially unreliable, or difficult to interpret automatically. Offshore investigations frequently encounter missing or poor-quality measurements due to equipment limitations, sensor malfunction, or environmental conditions. Under such circumstances, direct graphical interpretation cannot be consistently applied, whereas the proposed model can still provide a stable classification. In addition, the method enables automated interpretation for large CPT datasets, reducing manual interpretation effort and subjective variability among engineers. The model therefore serves as a decision-support tool rather than a replacement for established geotechnical procedures.
5. Nevertheless, several limitations should be acknowledged. The proposed model inherits the inherent uncertainty of the SBT classification system itself and does not explicitly quantify prediction uncertainty. As such, its outputs should be interpreted as supportive information within the broader geotechnical assessment framework.

## Data Availability

The data supporting the findings of this study can be obtained from the corresponding author upon reasonable request.
